# Bioptische Diagnostik ANCA‑assoziierter Vaskulitiden im HNO‑Trakt

**DOI:** 10.1007/s00393-022-01303-4

**Published:** 2022-12-21

**Authors:** Martin Laudien, Konstanze Holl-Ulrich

**Affiliations:** 1 Klinik für Hals‑, Nasen‑, Ohrenheilkunde, Kopf- und Halschirurgie, Christian-Albrechts-Universität zu Kiel und Universitätsklinikum Schleswig-Holstein, Campus Kiel, Arnold-Heller-Str. 3, Haus B1, 24105 Kiel, Deutschland; 2 Konsultations- und Referenzzentrum für Vaskulitis-Diagnostik, Pathologie – Hamburg, Labor Lademannbogen MVZ GmbH, Hamburg, Deutschland

Bei bis zu 80 % der Patient:innen kommt es im Laufe einer Granulomatose mit Polyangiitis (GPA) oder eosinophilen Granulomatose mit Polyangiitis (EGPA) zu einer Manifestation auf hals-, nasen-, ohrenärztlichem (HNO) Fachgebiet [6, 10]. Aktuelle wissenschaftliche Arbeiten ergeben Hinweise auf unterscheidbare Phänotypen mit distinktem klinischem Verlauf in Abhängigkeit einer solchen Manifestation [9].

Eine interdisziplinäre Behandlung und Nachuntersuchungen sind unabdingbar [13].

Die Untersuchungen für das HNO-Fachgebiet sollten, regelmäßig wiederholt, beinhalten:

- Inspektion des Integuments,
- Inspektion der Mundhöhle,
- Endoskopie der Mundhöhle, der Nasenhaupthöhle, des Epi-Oropharynx, Larynx und der subglottischen Region,
- Otoskopie,
- Funktionsdiagnostik (Hirnnerven insbesondere auch Olfaktometrie, Audiologie).

Bei Patient:innen mit mikroskopischer Polyangiitis (MPA) treten in aller Regel keine spezifischen Veränderungen im HNO-Trakt auf. Selten sind Fälle mit Rhinosinusitis und Epistaxis beschrieben [12]. Eine bioptische Diagnostik ist nicht zielführend.

Patient:innen mit eosinophiler Granulomatose mit Polyangiitis (EGPA) berichten regelmäßig über Beschwerden auf HNO-ärztlichem Fachgebiet, insbesondere sinusitische [8]. Meist lassen sich endoskopisch kontrolliert nasal Polypen detektieren. Eine Heilung der nasalen Manifestation durch eine chirurgische Therapie (funktionell endoskopische Nasennebenhöhlenchirurgie) ist in der überwiegenden Zahl der Fälle frustran, da die Imbalance der Störung der nasalen Grenzfläche hierdurch nur unzureichend zu beeinflussen ist. Bei Verdacht auf EGPA zeigt sich in der polypös veränderten Nasen- bzw. Nasennebenhöhlenschleimhaut meist eine interstitielle Eosinophilie, wie sie identisch auch bei allergischer Rhinitis bzw. Sinusitis ohne EGPA nachweisbar ist. Eine Vaskulitis oder eosinophile Granulome fehlen im HNO-Trakt bei EGPA in aller Regel, sodass eine EGPA im HNO-Trakt bioptisch nicht bewiesen werden kann. Dennoch können der Nachweis und die Quantifizierung der lokalen Eosinophilie eine Voraussetzung zur medikamentösen Therapie mit z. B. Biologicals darstellen. Mepolizumab hat jüngst eine Zulassung zur Behandlung bei chronisch polypöser Sinusitis erhalten, und so ist der Weg für einen „in label use“ geebnet. Bei der Interpretation der Gewebeeosinophilie muss ggf. die vorherige Gabe von Kortikosteroiden, auch lokal, berücksichtigt werden.

Die Manifestationen der GPA auf dem HNO-Fachgebiet können mit guter Inter- und Intraraterreabilität beurteilt und unter Zuhilfenahme des Ear‑, Nose- and Throat Activity Scores (ENTAS) standardisiert dokumentiert werden [2].

Die Schleimhaut der Nase und Nasennebenhöhlen stellt eine Prädilektionsstelle der Aktivität auf dem HNO-Gebiet dar und ist der Inspektion sowie Biopsie leicht zugänglich (Abb. 1). Des Weiteren kommt es unter anderem insbesondere hinsichtlich lebensbedrohlicher Manifestationen zu subglottischen Inflammationen/Vernarbungen.

**Figure 1:**
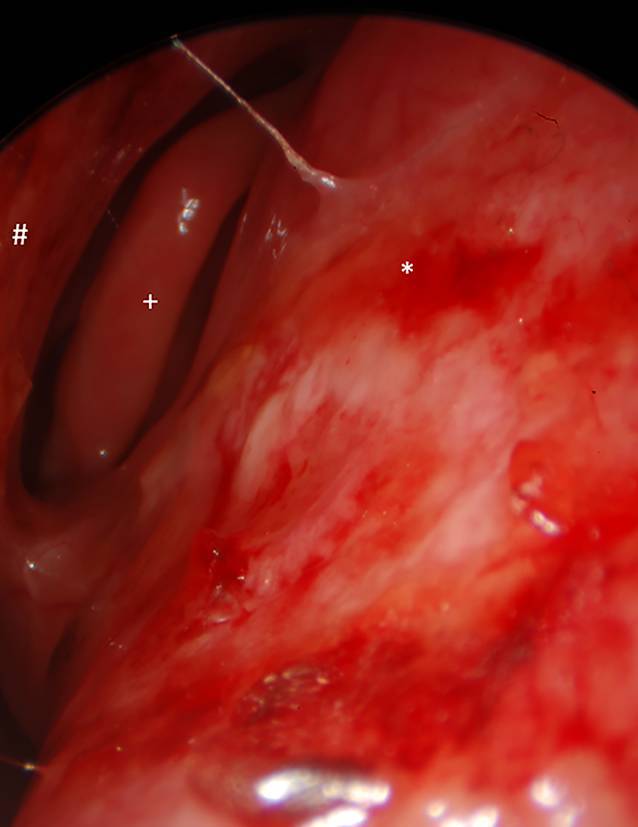

Neben den Schwierigkeiten der Unterscheidung zwischen nasaler Aktivität der Erkrankung, Funktionsverlust der Mukosa (mit Austrocknungen, Verkrustungen und konsekutiven rezidivierenden nasalen Blutungen), Infektion, Superinfektion, Schaden (nicht zu verwechseln mit Aktivität) durch die Erkrankung und/oder Therapie müssen Differenzialdiagnosen bedacht und regelmäßig im Verlauf evaluiert werden. Zu diesen Differenzialdiagnosen zählen insbesondere der Substanzmissbrauch (vor allem Kokain und hierbei besonders die Zusatzstoffe [5]), das nasale NK/T-Zell-Lymphom, die invasive Mykose oder Malignome wie das Plattenepithelkarzinom. Einer langjährigen Therapie mit hochpotenten Immunmodulatoren mag eine Bedeutung in der Entwicklung von (Ko‑)Erkrankungen zukommen [5, 11].

Der histopathologischen Diagnostik einer GPA kommt im HNO-Trakt eine hohe Bedeutung zu, da bei etwa der Hälfte der Fälle bei Erstmanifestation im HNO-Trakt kein Anti-Neutrophilen-zytoplasmatischer Antikörper (ANCA) nachweisbar ist [1]. Bei persistierender lokalisierter Erkrankung bleiben etwa 50 % dieser Patienten auch im Verlauf ANCA-negativ [3].

Biopsien müssen ausreichend groß und tief sein, um eine histopathologische Diagnosestellung zur ermöglichen (Abb. 2). Nach unserer Erfahrung hat sich die Entnahme von mehreren, jeweils mindestens 0,3 cm durchmessenden Biopsien bewährt. Hierbei ist es notwendig, erkranktes vitales Gewebe einschließlich der kleinen Gefäße zu erreichen. Dies gelingt besser aus dem Randbereich der Läsionen. Das Gewebe wird in 4 % gepuffertem Formalin fixiert übersandt. Die zusätzliche Entnahme von Frischmaterial für die mikrobiologische Diagnostik kann bei den genannten Differenzialdiagnosen sinnvoll sein. Jeder Entnahme sollten für die histopathologische Beurteilung stets klinische Angaben zur Aktivität, zum makroskopischen und serologischen Befund beigefügt werden (z. B. auch Kopie des letzten Arztbriefes).

**Figure 2:**
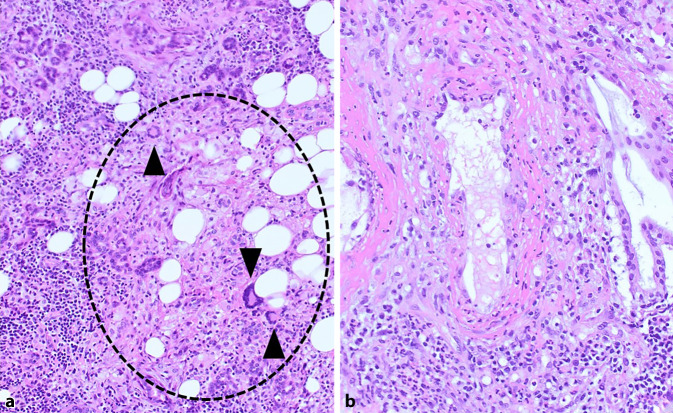

Bestimmte Lokalisationen sind leicht, für die Patient:innen wenig belastend und ohne wesentliche Komplikationsraten zu erreichen (z. B. die laterale Nasenwand), an anderen Lokalisationen ist die Indikation zur Biopsie eng zu stellen (z. B. subglottische Region, da weitere Vernarbungen der engsten Stelle der Luftwege schnell funktionelle Einschränkungen hervorrufen können).

Häufig gelingt der Nachweis charakteristischer Veränderungen einer GPA erst kumulativ aus wiederholten Entnahmen [4]. Hier kann die Nachbefundung evtl. bereits früher entnommenen Gewebes durch ein spezialisiertes Referenzzentrum hilfreich sein. Diagnostisch ergiebig sind insbesondere Schleimhautresektate aus (vorangegangenen) Nasennebenhöhlen- oder Mastoidoperationen. Hierbei sollte den Patholog:innen unbedingt mitgeteilt werden, ob bereits wiederholte Eingriffe vorausgegangen sind, da das histologische Bild von postoperativen Veränderungen und Fremdkörperreaktionen überlagert sein kann, die eine Abgrenzung zur GPA erschweren.

Weitere chirurgische Verfahren haben ihre Berechtigung bei Komplikationen (z. B.: Mukozelen, Dyspnoe, Visusverlust) und in der Rekonstruktion/Rehabilitation (z. B.: Septorhinoplastik, Trachearekonstruktion, Hörrehabilitation) [7].

## Fazit für die Praxis

Biopsien aus dem HNO-Trakt bei Verdacht auf GPA müssen aus makroskopisch auffälligen Arealen entnommen werden. Die Gewebsstücke müssen ausreichend groß, tief und evtl. repetitiv sein (möglichst mehrere Partikel von 0,3 cm Durchmesser). Die klinische und histopathologische Zweitbegutachtung vorheriger Resektate durch Referenzzentren kann in der Diagnostik einer GPA hilfreich sein. Bei EGPA unterscheidet sich das histopathologische Bild nicht von einer allergischen Rhinitis/Sinusitis ohne EGPA.
